# Supplementary material for: Beyond Trypanosoma cruzi: LINE-1 Activation as a Driver of Chronic Inflammation in Chagas Disease
Source: Int J Mol Sci. 2025 May 8;26(10):4466. doi: 10.3390/ijms26104466 (PMC12111687; doi:10.3390/ijms26104466)
Supplement: Supplementary file 1 [file ijms-26-04466-s001.zip › Table S1.pdf]

Table S1. Primer Sequences, Annealing Temperatures, Target Genes, and Associated Pathways for qPCR Analysis.

Gene	Sense	Primer Sequence	Annealing Temperature	Target	Pathway
**APOBEC-3**	Foward	5′GAGAAGGGACAAGCACATGG3′	60°C	HEK-293	L1 inhibition
	Reverse	5′TGGATCCATCAAGTGTCTGG3′			
**MOV-10**	Foward	5′GGCAAGACTGTCACGTTAGTGG3′	60°C	HEK-293	L1 inhibition
	Reverse	5′GGAGCCTTTGACAGAGTAGGTC3′			
**SAMDH-1**	Foward	5′CTCGCAACTCTTTACACCGTAGA3′	60°C	HEK-293	L1 inhibition
	Reverse	5′TTTCCTCCAGCACCTGTAATCTC3′			
**TREX-1**	Foward	5′CCACTCCTTTCCTTACCACATC3′	60°C	HEK-293	L1 inhibition
	Reverse	5′CCACTCCGCCAAACAGAT3′			
**LINE-1**	Foward	5′ACACCTATTCCAAAATTGACCAC3′	63°C	HEK-293	-
	Reverse	5′TTCCCTCTACACACTGCTTTGA3′			
**ATM**	Foward	5′TGTTCCAGGACACGAAGGGAGA3′	60°C	HEK-293	Homologous recombination
	Reverse	5′CAGGGTTCTCAGCACTATGGGA3′			
**MSH-2**	Foward	5′CAGCAGTCAGAGCCCTTAACCT3′	60°C	HEK-293	Mismatch repair
	Reverse	5′GAGAGGCTGCTTAATCCACTGG3′			
**OGG-1**	Foward	5′GGCTCAACTGTATCACCACTGG3′	60°C	HEK-293	Base excision repair
	Reverse	5′GGCGATGTTGTTGTTGGAGGAAC3′			
**XPA**	Foward	5′GAAGTCCGACAGGAAAACCGAG3′	60°C	HEK-293	Nucleotide excision repair
	Reverse	5′GATGAACAATCGTCTCCCTTTTCC3′			
**XRCC-4**	Foward	5′ATGGCTCCTCAGGAGAATCAGC3′	60°C	HEK-293	Non-homologous end joining
	Reverse	5′GAGGTCTTCTGGGCTGCTGTTT3′			
**RIG-1**	Foward	5′GGTTTAGGGAGGAAGAGGTGC3′	58°C	HEK-293	RNA sensor
	Reverse	5′AAGTGTGGCAGCCTCCATTG3′			
**MDA-5**	Foward	5′GCTGAAGTAGGAGTCAAAGCCC3′	60°C	HEK-293	RNA sensor
	Reverse	5′CCACTGTGGTAGCGATAAGCAG3′			
**IFN-α**	Foward	5′TGGGCTGTGATCTGCCTCAAAC3′	60°C	HEK-293	Interferon
	Reverse	5′CAGCCTTTTGGAACTGGTTGCC3′			
**IFN-β**	Foward	5′CTTGGATTCCTACAAAGAAGCAGC3′	60°C	HEK-293	Interferon
	Reverse	5′TCCTCCTTCTGGAACTGCTGCA3′			
**IFN-γ**	Foward	5′ACTGTCGCCAGCAGCTAAAA3′	56°C	HEK-293	Interferon
	Reverse	5′TATTGCAGGCAGGACAACCA3′			
**TBP**	Foward	5′TGTATCCACAGTGAATCTTGGTTG3′	60°C	HEK-293	Constitutive
	Reverse	5′GGTTCGTGGCTCTCTTATCCTC3′			

**IFN-α**	Foward	5′GGATGTGACCTTCCTCAGACTC3′	60°C	*Mus musculus*	Interferon
	Reverse	5′ACCTTCTCCTGCGGGAATCCAA3′			
**IFN-β**	Foward	5′GCCTTTGCCATCCAAGAGATGC3′	58°C	*Mus musculus*	Interferon
	Reverse	5′ACACTGTCTGCTGGTGGAGTTC3′			
**IFN-γ**	Foward	5′GCCCAATATCTCGGATGCTTC3′	60°C	*Mus musculus*	Interferon
	Reverse	5′GCCAAAATAGCTTCGGTAATCCT3′			
**LINE-1**	Foward	5′TTGGCGTGACTCTAACTAAGGAG3′	58°C	*Mus musculus*	-
	Reverse	5′CCTAGGTTTTTTGTTATTCCAGACA3′			
**β-actina**	Foward	5′CGTACTCCTGCTTGCTGATCCACATC TGC3′	60°C	*Mus musculus*	Constitutive
	Reverse	5′ATCTGGCACCACACCTTCCTACAAT GAGCTGCG3′			
**Tc40S**	Foward	5′TGCGAAGACGAGGAGTACAA3′	58°C	*Trypanosoma cruzi*	Constitutive
	Reverse	5′GCCACACACGAGCACTTAAA3′			
